# Supplementary material for: Isoprene-Degrading Bacteria from Soils Associated with Tropical Economic Crops and Framework Forest Trees
Source: Microorganisms. 2021 May 10;9(5):1024. doi: 10.3390/microorganisms9051024 (PMC8150984; doi:10.3390/microorganisms9051024)
Supplement: Supplementary file 1 [file microorganisms-09-01024-s001.zip › microorganisms-1211570-supplementary.pdf]

Supplementary Materials

Isoprene-Degrading Bacteria from Soils Associated with Tropical Economic Crops and Framework Forest Trees

Toungporn Uttarotai, Boyd A. McKew, Farid Benyahia, J. Colin Murrell, Wuttichai Mhuantong, Sunanta Wangkarn, Thararat Chitov, Sakunnee Bovonsombut, and Terry J. McGenity

Table S1. Soil type and mean values of temperature, moisture and pH, n = 5.

Plant Species	Soil type	Soil temperature (°C)			Soil moisture (% dry weight)			pH		
		rainy	winter	summer	rainy	winter	summer	rainy	winter	summer
Council tree	Clay loam	19.0	17.0	20.0	41.30	39.10	36.54	5.3	5.4	5.5
Wild oak	Clay loam	19.0	17.0	21.0	44.05	38.29	37.10	5.2	5.3	5.3
Wild Himalayan cherry	Clay loam	19.5	17.5	20.0	43.18	35.14	33.27	5.3	5.2	5.3
Oil palm	Sandy loam	23.0	22.0	26.0	26.20	21.11	14.60	5.7	5.8	5.8
Rubber tree	Sandy loam	24.0	22.5	26.0	25.08	20.89	13.03	5.6	5.9	5.7
Sugar cane	Sandy loam	23.0	22.5	25.0	22.34	19.52	12.95	5.8	5.7	5.8

Table S2. Identification of isoprene-degrading isolates based on partial 16S rRNA gene sequence analysis

Source	Isolate	Accession number	Closest relative	% Identity	Accession number
Wild Himalayan cherry	17f	MW509984	*Ochrobactrum oryzae* strain NBRC 102588	99.32	NR114151
Wild Himalayan cherry	z29	MW509985	*Arthrobacter woluwensis* strain 1551	98.72	NR044894
Wild Himalayan cherry	32f	MW509983	*Bacillus frigoritolerans* strain DSM 8801	99.72	NR115064
Wild Himalayan cherry	39f	MW509987	*Friedmanniella spumicola* strain Ben 107	95.77	NR024907
Wild oak	z19	MW509988	*Klebsiella oxytoca* strain JCM1665	99.57	NR112010
Sugar cane	m29	MW509981	*Bacillus aryabhattai* strain B8W22	99.86	NR115953
Sugar cane	17	MW509982	*Isoptericola nanjingensis* strain H17	99.78	NR117941
Oil palm	23	MW509986	*Cellulosimicrobium funkei* strain W6122	99.86	NR042937
